# Supplementary material for: Virus-associated anterior uveitis and secondary glaucoma: Diagnostics, clinical characteristics, and surgical options
Source: PLoS One. 2020 Feb 24;15(2):e0229260. doi: 10.1371/journal.pone.0229260 (PMC7039515; doi:10.1371/journal.pone.0229260)
Supplement: S1 Table — (DOCX) [file pone.0229260.s001.docx]

**Table S1.** Summary of immune mediators’ levels (pg/mL) in log10

| **Immune mediators** | **CMV (n=23)** | **HSV (n=34)** | **VZV (n=16)** | **RV (n=77)** | **p-Value** |
| --- | --- | --- | --- | --- | --- |
|  | Median (Range) | Median (Range) | Median (Range) | Median (Range) |  |
| IL-1b | 0.27 (0-1.13) | 0.06 (0-2.11) | 0.62 (0-1.88) | 0 (0-1.2) | 0.5032 |
| IL-1RA | 1.77 (0-2.95) | 0.62 (0-2.8) | 1.34 (0-3.21) | 0.81 (0-2.53) | 0.0932 |
| IL-2 | 0 (0-2.62) | 0 (0-2.55) | 0 (0-2.57) | 0.26 (0-2.37) | 0.5249 |
| IL-4 | 0 (0-1.81) | 0 (0-1.92) | 0 (0-1.89) | 0 (0-1.67) | 0.8396 |
| IL-5 | 0 (0 – 1.74) | 0 (0-2.65) | 0 (0-2.73) | 0.52 (0-2.03) | 0.2885 |
| IL-6 | 2.31 (0-4.39) | 2.64 (0-5.39) | 3.17 (0-4.4) | 2.77 (0-4.21) | 0.6081 |
| IL-7 | 2.77 (0-3.05) | 0.34 (0-3.29) | 0.72 (0-3.32) | 2.8 (0-3.23) | 0.2807 |
| IL-8 | 1.81 (0-3.13) | 1.48 (0-3.08) | 1.27 (0-2.96) | 1.82 (0-2.98) | 0.3139 |
| IL-9 | 1.32 (0-2.47) | 0 (0-2.61) | 0 (0-2.74) | 0.12 (0-2.45) | 0.1483 |
| IL-10 | 1.14 (0-2.63) | 0.58 (0-1.95) | 0.42 (0-3.23) | 0.41 (0-2.51) | 0.1157 |
| **IL-12** | 2.12 (0.49-2.74) | 1.4 (0-2.59) | 1.59 (0-2.81) | 2.21 (0-2.71) | **0.0149** |
| IL-13 | 2.17 (0-3.02) | 0.18 (0-3.06) | 0 (0-3.23) | 2.35 (0-3.39) | 0.1170 |
| **IL-15** | 1.41 (0-2.29) | 1.06 (0-2.18) | 0.92 (0-2.34) | 1.61 (0-2.26) | **0.0249** |
| IL-17 | 0 (0-2.65) | 0 (0-2.77) | 0 (0-2.95) | 0 (0-2.39) | 0.0815 |
| **Eotaxin** | 2.778 (0-3.15) | 0.57 (0-3.03) | 1.02 (0-3.08) | 1.61 (0-3.03) | **0.0215** |
| FGFbasic | 0.94 (0-18) | 0.93 (0-17) | 0.82 (0-1.88) | 0.65 (0-1.61) | 0.3586 |
| G-CSF | 1.93 (0-3.97) | 0 (0-4.96) | 2.05 (0-3.6) | 1.87 (0-3.79) | 0.4442 |
| GM-CSF | 0 (0-2.19) | 0.44 (0-2.03) | 0.89 (0-2.47) | 0 (0-1.99) | **0.0059** |
| IFNg | 1.69 (0-3.21) | 0.67 (0-3.13) | 1.57 (0-3.76) | 1.31 (0-2.83) | 0.3231 |
| **IP-10** | 4.92 (2.96-5.97) | 4.63 (2.29-6.24) | 4.51 (2.48-5.95) | 4.76 (2.64-6.41) | **0.0010** |
| **MCP1** | 2.99 (1.71-3.67) | 2.82 (0-3.72) | 2.59 (1.56-3.82) | 3.15 (1.73-3.74) | **0.0078** |
