# Supplementary figures and images for: Virus-associated anterior uveitis and secondary glaucoma: Diagnostics, clinical characteristics, and surgical options
Source: PLoS One. 2020 Feb 24;15(2):e0229260. doi: 10.1371/journal.pone.0229260 (PMC7039515; doi:10.1371/journal.pone.0229260)

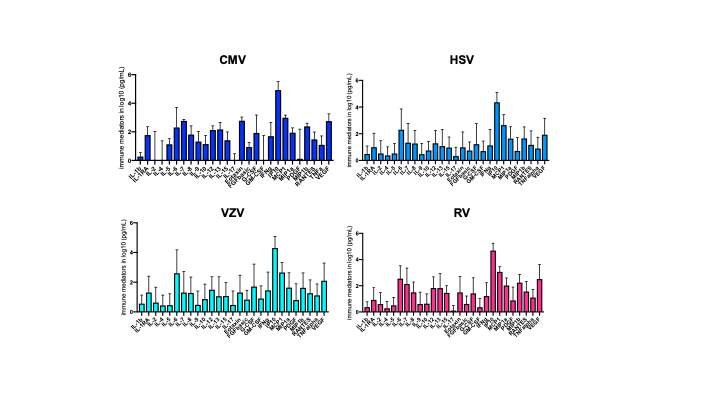

Supplement: S1 Fig — (TIFF) [file pone.0229260.s002.tiff]
