# Supplementary material for: Virus-associated anterior uveitis and secondary glaucoma: Diagnostics, clinical characteristics, and surgical options
Source: PLoS One. 2020 Feb 24;15(2):e0229260. doi: 10.1371/journal.pone.0229260 (PMC7039515; doi:10.1371/journal.pone.0229260)
Supplement: Attachment — Submitted filename: Response_to_Reviewers-Virus_PlosONE_3.docx [file pone.0229260.s004.docx]

Charité ⏐ Campus Virchow-Klinikum ⏐ 13353 Berlin

**James T. Rosenbaum, MD**

**Editor-in-Chief**

**PLOS ONE**

**Department of Ophthalmology**

**Prof. Dr. med. U. Pleyer**

Direktor: Prof. Dr. med. A. Joussen

Tel.: 030/450 654131

Fax: 030/450 554900

domika.pohlmann@charite.de

Nov 29^th^ 2019

**Dear Prof. Rosenbaum,**

**Dear Prof. Awadein,**

**Dear Reviewers,**

We sincerely thank you for your constructive and astute remarks on our manuscript and your thoughtful recommendations. We have taken the opportunity to carry out substantial enhancements.

We hope now that all issues are well processed.

We also uploaded an anonymized data set to replicate our study findings as Supporting Information files. We accept to share the data. For further queries Uly Sumarni can be contacted (uly.sumarni@googlemail.com).

We uploaded a new Figure 2.

All authors have approved this revised version and hope that you now find it acceptable for publication.

Kind regards,

Dominika Pohlmann, MD and Milena Pahlitzsch, MD

**Manuscript “Virus-associated anterior uveitis and secondary glaucoma:**

**Diagnostics, clinical characteristics, and surgical options ”**

Ref: PONE-D-19-29232

Explanations and changes of the revised manuscript 16/11/2019

| **Reviewer's comments** | **Response** | **Changes in the manuscript** |
| --- | --- | --- |
| **Journal Requirements:** | | |
| Please ensure that your manuscript meets PLOS ONE's style requirements, including those for file naming. | We have made changes according to the PLOS ONE´s style requirements. |  |
| Please include in your financial disclosure statement the name of the funders of this study (as well as grant numbers if available). At present, this information is only available in the funding section of your manuscript. | This study was not funded.  Dr. Dominika Pohlmann is participant in the BIH Charité Clinician Scientist Program funded by the Charité- Universitätsmedizin Berlin and the Berlin Institute of Health.  We acknowledge support from the German Research Foundation (DFG) and the Open Access Publication Fund of Charite ́ – Universitätsmedizin Berlin. |  |
| In the ethics statement in the manuscript and in the online submission form, please provide additional information about the patient records and samples used in your retrospective study. Specifically, please ensure that you have discussed whether all data were fully anonymized before you accessed them and/or whether the IRB or ethics committee waived the requirement for informed consent. If patients provided informed written consent to have data from their medical records used in research, please include this information. | Data were fully anonymized before analysis and thus following German legal requirements it is not necessary that the ethics committee accesses a retrospective study design. | Page 3:  The patients gave informed consent before anterior chamber (AC) punction and glaucoma surgery. The following clinical characteristics were collected from patient´s medical records before the AC punction and in patients who underwent glaucoma surgery pre- and postoperatively:  Page 4:  The AH samples were immediately processed after the anterior chamber punction. |
| Thank you for stating the following in the Acknowledgments Section of your manuscript:   Dr. Dominika Pohlmann is participant in the BIH Charité Clinician Scientist Program funded by the Charité- Universitätsmedizin Berlin and the Berlin Institute of Health. The authors are free of a financial interest.  We note that you have provided funding information that is not currently declared in your Funding Statement. However, funding information should not appear in the Acknowledgments section or other areas of your manuscript. We will only publish funding information present in the Funding Statement section of the online submission form.  Please remove any funding-related text from the manuscript and let us know how you would like to update your Funding Statement. Currently, your Funding Statement reads as follows:   The funders had no role in study design, data collection and analysis, decision to publish, or preparation of the manuscript. | I stated the funding in the funding section.  The authors received no specific funding for this work. |  |
| We note that you have indicated that data from this study are available upon request. PLOS only allows data to be available upon request if there are legal or ethical restrictions on sharing data publicly. For information on unacceptable data access restrictions, please see http://journals.plos.org/plosone/s/data-availability#loc-unacceptable-data-access-restrictions. In your revised cover letter, please address the following prompts: a) If there are ethical or legal restrictions on sharing a de-identified data set, please explain them in detail (e.g., data contain potentially identifying or sensitive patient information) and who has imposed them (e.g., an ethics committee). Please also provide contact information for a data access committee, ethics committee, or other institutional body to which data requests may be sent. b) If there are no restrictions, please upload the minimal anonymized data set necessary to replicate your study findings as either Supporting Information files or to a stable, public repository and provide us with the relevant URLs, DOIs, or accession numbers. Please see http://www.bmj.com/content/340/bmj.c181.long for guidelines on how to de-identify and prepare clinical data for publication. For a list of acceptable repositories, please see http://journals.plos.org/plosone/s/data-availability#loc-recommended-repositories. We will update your Data Availability statement on your behalf to reflect the information you provide. | We uploaded anonymized and minimized data set to replicate our study findings as Supporting Information files.  We accept to share our anonymized and minimized data. |  |
| We note that you have included the phrase “data not shown” in your manuscript. Unfortunately, this does not meet our data sharing requirements. PLOS does not permit references to inaccessible data. We require that authors provide all relevant data within the paper, Supporting Information files, or in an acceptable, public repository. Please add a citation to support this phrase or upload the data that corresponds with these findings to a stable repository (such as Figshare or Dryad) and provide and URLs, DOIs, or accession numbers that may be used to access these data. Or, if the data are not a core part of the research being presented in your study, we ask that you remove the phrase that refers to these data. | We deleted this phrase. |  |
| **Reviewer #1** | | |
| I congratulate the authors for a well written and clear manuscript and a well designed study. The tables are very well presented although some table are rather exhaustive The results are very informative and relevant to the clinical practice | Thank you very much for your detailed view of our manuscript. We agree that this paper is relevant for starting therapeutic regimes in the virus associated anterior Uveitis. |  |
| **Reviewer #2** | | |
| The study question and idea is interesting and addressing an important gap in literature , the manuscript is also well written some points however still need to be addressed: | Thank you for your appreciation of our manuscript. We hope that we can fill some gaps in the current literature. We also added in the acknowledgment section that the abstract was accepted and presented as oral presentation at the International Ocular Inflammation Society Congress in Taiwan on November 13-16^th^ 2019. |  |
| 1. the number of patients mentioned throughout the manuscript is 270 patients (=eyes), this is unclear as the authors mentioned in table (1) that 40 cases were bilateral | This point is well taken. The bilateral involvements refer to a keratitis, but not a keratouveitis. We performed the AC tap only in one eye. In order to prevent misunderstanding, we have re-written the sentence in a more comprehensible way. | Page 6: To note, the second eye presented only a keratitis, not a keratouveitis, |
| 2. in table (1) : the authors mentioned that 123 case experienced an acute course of uveitis (hypertensive uveitis) , It is not understood why operation was needed during acute iridocyclitis which most cases can be temporarily controlled with medical treatment till remission occurs , even though in the rare circumstances where urgent surgery is needed , it is very difficult to judge whether the IOP has been surgically controlled or has it been the natural course of the disease . also evaluation of the success rate of surgical intervention in these cases is highly variable and can cause a statistical bias. | Thank you very much for your detailed view.  We did not perform any surgical therapy during an acute course of uveitis. We always started surgery under controlled inflammatory conditions as stated in the method section.  Of course you are right, and we would not know the course of natural history, when we would do acute surgical interventions.  We defined the acute course of inflammation from onset until a period of three weeks` time. | Page 5:  Surgical interventions were always conducted under stable inflammatory conditions. |
| 3. the surgical success rate was not clearly defined and the number of acute attacks could have been statistically correlated with the IOP control | Thank you very much for this comment.  In the revised version we stated a more detailed version of success rate which might differ in individuals, especially in uveitis cohort.  Success rate in surgical glaucoma papers is usually defined by the criteria of Kaplan Maier analysis.  This statistical method could not be performed due to our small number of interventions.  [*Gabbay IE*](https://www.ncbi.nlm.nih.gov/pubmed/?term=Gabbay%20IE%5BAuthor%5D&cauthor=true&cauthor_uid=31727624)*,* [*Allen F*](https://www.ncbi.nlm.nih.gov/pubmed/?term=Allen%20F%5BAuthor%5D&cauthor=true&cauthor_uid=31727624)*,* [*Morley C*](https://www.ncbi.nlm.nih.gov/pubmed/?term=Morley%20C%5BAuthor%5D&cauthor=true&cauthor_uid=31727624)*,* [*Pearsall T*](https://www.ncbi.nlm.nih.gov/pubmed/?term=Pearsall%20T%5BAuthor%5D&cauthor=true&cauthor_uid=31727624)*,* [*Bowes OM*](https://www.ncbi.nlm.nih.gov/pubmed/?term=Bowes%20OM%5BAuthor%5D&cauthor=true&cauthor_uid=31727624)*,* [*Ruben S*](https://www.ncbi.nlm.nih.gov/pubmed/?term=Ruben%20S%5BAuthor%5D&cauthor=true&cauthor_uid=31727624)*. Efficacy and safety data for the XEN45 implant at 2 years: a retrospective analysis.* [*Br J Ophthalmol.*](https://www.ncbi.nlm.nih.gov/pubmed/31727624) *2019 Nov 14.* | Page 5:  Success rate is defined by a controlled IOP and inflammatory situation which might differ in individuals due to their severity of glaucomatous damage. |
| 4. It is not clear why the study is retrospective although the follow up plan is clearly defined in page 11 "...Patients attended clinics preoperatively as well as follow-ups one day, six weeks, three months, six months, one year, and two years postoperatively." | Thank you very much for this comment. We are happy to have established this follow up schedule for all our glaucoma surgeries in our clinic. Thus, all patients get appointments for these follow ups before they leave the hospital. |  |
| 5. In table 4 : 2 cases needed CPC , this is needes further elaboartion as CPC in uveitic glaucoma is not favourable being associated with high incidence of pthisis bulbi so please outline the points of view in taking such decision | Thank you for your comment:  To our knowledge CPC is not significantly associated with an increased rate of phtisis bulbi. We perform CPC frequently in secondary glaucoma, if necessary, and did not notice an increased rate of phtisis. The most important risk factor for a phtisis is probably the applied laser energy and might be a reason why some centers will experience side effects of the CPC in uveitic glaucoma.  Comparative research studies:   1. [*Kopel J*](https://www.ncbi.nlm.nih.gov/pubmed/?term=Kopel%20J%5BAuthor%5D&cauthor=true&cauthor_uid=7738290)*,* [*Valtot F*](https://www.ncbi.nlm.nih.gov/pubmed/?term=Valtot%20F%5BAuthor%5D&cauthor=true&cauthor_uid=7738290)*,* [*Poirier C*](https://www.ncbi.nlm.nih.gov/pubmed/?term=Poirier%20C%5BAuthor%5D&cauthor=true&cauthor_uid=7738290)*,* [*Moulin F*](https://www.ncbi.nlm.nih.gov/pubmed/?term=Moulin%20F%5BAuthor%5D&cauthor=true&cauthor_uid=7738290)*,* [*Morel C*](https://www.ncbi.nlm.nih.gov/pubmed/?term=Morel%20C%5BAuthor%5D&cauthor=true&cauthor_uid=7738290)*,* [*Haut J*](https://www.ncbi.nlm.nih.gov/pubmed/?term=Haut%20J%5BAuthor%5D&cauthor=true&cauthor_uid=7738290)*.[Treatment of refractory glaucoma with Nd:YAG laser cyclophotocoagulation].* [*J Fr Ophtalmol.*](https://www.ncbi.nlm.nih.gov/pubmed/?term=phtisis+cyclophotocoagulation) *1995;18(1):13-21.* 2. [*Voykov B*](https://www.ncbi.nlm.nih.gov/pubmed/?term=Voykov%20B%5BAuthor%5D&cauthor=true&cauthor_uid=24390401)*,* [*Deuter C*](https://www.ncbi.nlm.nih.gov/pubmed/?term=Deuter%20C%5BAuthor%5D&cauthor=true&cauthor_uid=24390401)*,* [*Zierhut M*](https://www.ncbi.nlm.nih.gov/pubmed/?term=Zierhut%20M%5BAuthor%5D&cauthor=true&cauthor_uid=24390401)*,* [*Leitritz MA*](https://www.ncbi.nlm.nih.gov/pubmed/?term=Leitritz%20MA%5BAuthor%5D&cauthor=true&cauthor_uid=24390401)*,* [*Guenova E*](https://www.ncbi.nlm.nih.gov/pubmed/?term=Guenova%20E%5BAuthor%5D&cauthor=true&cauthor_uid=24390401)*,* [*Doycheva D*](https://www.ncbi.nlm.nih.gov/pubmed/?term=Doycheva%20D%5BAuthor%5D&cauthor=true&cauthor_uid=24390401)*.Is cyclophotocoagulation an option in the management of glaucoma secondary to Fuchs' uveitis syndrome?* [*Graefes Arch Clin Exp Ophthalmol.*](https://www.ncbi.nlm.nih.gov/pubmed/24390401) *2014 Mar;252(3):485-9.* 3. [Aujla JS](https://www.ncbi.nlm.nih.gov/pubmed/?term=Aujla%20JS%5BAuthor%5D&cauthor=true&cauthor_uid=23433313), [Lee GA](https://www.ncbi.nlm.nih.gov/pubmed/?term=Lee%20GA%5BAuthor%5D&cauthor=true&cauthor_uid=23433313), [Vincent SJ](https://www.ncbi.nlm.nih.gov/pubmed/?term=Vincent%20SJ%5BAuthor%5D&cauthor=true&cauthor_uid=23433313), [Thomas R](https://www.ncbi.nlm.nih.gov/pubmed/?term=Thomas%20R%5BAuthor%5D&cauthor=true&cauthor_uid=23433313). Incidence of hypotony and sympathetic ophthalmia following trans-scleral cyclophotocoagulation for glaucoma and a report of risk factors. [Clin Exp Ophthalmol.](https://www.ncbi.nlm.nih.gov/pubmed/23433313) 2013 Nov;41(8):761-72. |  |
| 6. At which point was the "Goldmann-Witmer " test done? during the attack of AAU or in CAU ? and how sensitive/ specific this test is | As a tertiary referral center the patients are referred to us and we did not know the 100% accurate onset of the disease. But the baseline data (Table 1) show the disease course of point in time of the AC tap. In total, 123 (46%) eyes had an acute disease onset and 147 (54%) presented a course of disease lasting over a period of three weeks.  The Goldman-Witmer Coefficient analysis is well established diagnostic method in infectious uveitis. The colleagues from Netherland have already published many papers using this technique.  *Groen-Hakan F, Babu K, Tugal-Tutkun I et al. Challenges of Diagnosing Viral Anterior Uveitis. Ocul Immunol Inflamm. 2017;25(5):710-20.*  *Wensing B, Relvas LM, Caspers LE, et al. Comparison of rubella virus- and herpes virus associated anterior uveitis: clinical manifestations and visual prognosis. Ophthalmology. 2011;118(10):1905-1910.*  But unfortunately, there is only one paper about sensitivity and specificity of the test for toxoplasmosis in the literature.  *Fekkar A, Bodaghi B, Touafek F, et al. Comparison of immunoblotting, calculation of the Goldmann-Witmer coefficient, and real-time PCR using aqueous humor samples for diagnosis of ocular toxoplasmosis. J Clin Microbiol. 2008;46(6):1965-7.* |  |
| 7. the conclusion "MIGS can be used as first-line-treatment in individual cases, however, the device needs to be carefully chosen by experienced specialists based on the individual needs of the patient. Filtrating glaucoma surgery remains the gold standard" can not be drawn as a fact from this study as it needs prospective evaluation and standardization of patients criteria and severity of glaucoma to be properly evaluated | Thank you very much for this detailed comment.  Of course, you are right and a definition of gold standard cannot be stated by our retrospective data.  We have re-written the conclusion in the revised version of the manuscript. | Page 2: MIGS can be suggested as first-line-treatment in individual cases, however, the device needs to be carefully chosen by experienced specialists based on the individual needs of the patient. Filtrating glaucoma surgery can be recommended in VAU as an effective therapy to reduce the IOP over a longer period of time. |
